# Kinome-wide identification of phosphorylation networks in eukaryotic proteomes

**DOI:** 10.1093/bioinformatics/bty545

**Published:** 2018-07-17

**Authors:** Luca Parca, Bruno Ariano, Andrea Cabibbo, Marco Paoletti, Annalaura Tamburrini, Antonio Palmeri, Gabriele Ausiello, Manuela Helmer-Citterich

**Affiliations:** Department of Biology, Centro di Bioinformatica Molecolare, University of Rome “Tor Vergata”, Rome, Italy

## Abstract

**Motivation:**

Signaling and metabolic pathways are finely regulated by a network of protein phosphorylation events. Unraveling the nature of this intricate network, composed of kinases, target proteins and their interactions, is therefore of crucial importance. Although thousands of kinase-specific phosphorylations (KsP) have been annotated in model organisms their kinase-target network is far from being complete, with less studied organisms lagging behind.

**Results:**

In this work, we achieved an automated and accurate identification of kinase domains, inferring the residues that most likely contribute to peptide specificity. We integrated this information with the target peptides of known human KsP to predict kinase-specific interactions in other eukaryotes through a deep neural network, outperforming similar methods. We analyzed the differential conservation of kinase specificity among eukaryotes revealing the high conservation of the specificity of tyrosine kinases. With this approach we discovered 1590 novel KsP of potential clinical relevance in the human proteome.

**Availability and implementation:**

http://akid.bio.uniroma2.it

**Supplementary information:**

Supplementary data are available at *Bioinformatics* online.

## 1 Introduction

Protein phosphorylation is a fast and reversible regulation mechanism able to control the function and to regulate the activity of proteins and their interactions in signaling and metabolic pathways (Newman *et al.*, 2013; Oliveira *et al.*, 2012; Reimand and Bader, 2013; Sacco *et al.*, 2012; Schwammle *et al.*, 2014; Seet *et al.*, 2006). This post-translational modification is managed by a number of domains that have been selected through evolution to ‘write’ (through kinases), ‘erase’ (through phosphatases) and ‘read’ phosphorylations (Seet *et al.*, 2006) (e.g. the SH2 domain). Kinases recognize specific key proteins and their specific interactions are rewired in cancer-related signaling pathways (Creixell *et al.*, 2015a, b; Ferrè *et al.*, 2014; Palmeri *et al.*, 2014). Alterations of signaling networks with somatic mutations altering phosphorylation sites have been shown to be present in the majority of tumors and to involve known cancer genes with high conservation and functional impact (Reimand *et al.*, 2013). Among all the types of protein post-translational modification, protein phosphorylation is currently the most studied with more than 150 000 known modified protein residues (Minguez *et al.*, 2015). Around 500 human protein kinases have been characterized (Manning *et al.*, 2002b) and analyzed to find common and specific traits in their mode of action (Creixell *et al.*, 2015a; Mok et al., 2010; Zhu *et al.*, 2005). Different approaches, based on different sources of information (e.g. protein–protein interactions, gene expression profiling and genetic interactions) and methodologies (e.g. protein microarray) (Breitkreutz *et al.*, 2010; Fiedler *et al.*, 2009; Linding *et al.*, 2007; Newman *et al.*, 2013; Van Wageningen *et al.*, 2010), have been developed for the high-throughput identification of kinase-substrate relationships. Despite these efforts and the amount of data generated, the network connecting kinases and their target proteins through phosphorylation in a kinase-specific fashion is mostly unknown, with only 9159 known kinase-specific phosphorylations (KsP) in *Homo sapiens* (Hornbeck *et al.*, 2015). Moreover the numbers of annotated kinases and kinase-specific targets decrease in less studied and annotated organisms.

Different kinase-independent and kinase-specific methods have been developed to complement experimental approaches, trying to overcome their technical/biological limitations, in order to predict phosphorylation sites, using evolutionary, sequence and structure features elaborated with different approaches (e.g. Hidden Markov Models (HMMs), Support Vector Machines, Bayesian decision theory, Neural Networks), for example NetworKIN (Horn *et al.*, 2014), Musite Deep (Wang *et al.*, 2017), GPS 2.0 (Xue *et al.*, 2008), iGPS (Song *et al.*, 2012) and NetPhosK (Blom *et al.*, 2004). Although different in terms of availability and capability (e.g. kinase coverage, input size, methodology and execution time etc.), the majority of the methods characterize peptides known to be targeted by specific kinases using different features (e.g. amino-acid composition, sequence similarity, secondary structure, local charge state, solvent accessibility, coexpression and protein–protein interaction data). Kinases, and/or kinase families are generally used to label and group their target peptides in order to build a predictive kinase-specific, or kinase family-specific, model. An alternative approach is presented by the Predikin method (Ellis and Kobe, 2011) which takes into account the sequence of kinases, structural features of key residues, termed ‘determinants’, identified by analyzing the structure of a small set of kinases-target complexes and substrate specificities determined by peptide library experiments. In this way the kinase determinants are used to classify specific targets for each kinase. However the *a priori* knowledge of the kinase specificity rules is missing from the vast majority of the other methods.

Generally, these available methods rely on the assumption that signaling interactions and kinase specificities rules are conserved and shared in eukaryotes (Manning *et al.*, 2002a) in order to predict novel interactions in different organisms. These methods are usually trained on one or few eukaryotes with the highest amount of experimental data, in terms of kinases and known phosphosites, and then applied to other organisms. Organism-specific variations in the kinase specificity rules are expressed usually by kinase-specific groups of phosphosites that are used in the training phase leaving the kinase sequence out and therefore limiting the reach of a method. This would require the complete annotation of kinases for each species, which is a hard task especially in uncharacterized organisms.

With this in mind, we designed a method for the Automatic Kinase-specific Interaction Detection (AKID) that merges the information encoded in the kinase with the information encoded in the target peptide. AKID is capable of automatically detecting protein kinases in a given proteome (without any prior annotation), their key residues for specificities, termed Determinants of Specificity (DoS) and ultimately their target KsP sites. Differently from other already available methods, AKID does not require prior kinase annotation and uses the Kinspect (Creixell *et al.*, 2015a) approach, which does not rely on structural data, to capture kinase DoS, which are not necessarily in close contact with the binding site of the target peptide. In this work we trained the proposed approach on a curated training set of human KsP and tested it on proteomes belonging to different organisms comparing the performance with other published methods. We observed different performances of the method on the different kinase groups, which we examined in depth from an evolutionary perspective with the analysis of orthologous KsP in 45 different eukaryotes. Finally we show how the method can be used to discover novel KsP of potentially high clinical relevance in the human proteome.

We envision that these predicted KsP can shed more light on the mechanisms underlying pathogenic conditions. In order to allow an easy access of the method to researchers we developed a web interface and a standalone software (available at http://akid.bio.uniroma2.it).

## 2 Materials and methods

### 2.1 Identification of kinase domains

In order to identify the kinase domains in a given proteome, we used the hmmer (Finn *et al.*, 2015) package to build a HMM profile from a manually curated MSA of 530 human kinase domain sequences (Creixell *et al.*, 2015a). We used this HMM to identify all the kinase domains in the canonical proteome of all selected organisms (as detailed below) obtained from Ensembl version 79. We used the hmmscan function to scan the proteomes and identify the kinase domains as the matches with *P*-value <0.05.

### 2.2 Identification of the kinase determinants of specificity

We used the Kinspect method (Creixell *et al.*, 2015a) to identify the residues that are involved in determining the peptide specificity across the kinases. By analyzing an alignment of human kinase domains the authors of Kinspect were able to identify, and experimentally validate, the subset of residues that better account for the target peptide recognition (DoS) using a learning classifier system. The alignment of the human kinases, mentioned above, was analyzed using Kinspect, highlighting 63 DoS residues. The kinase domains that are identified in any eukaryotic proteome through the HMM are then individually aligned to the same HMM using the hmmer package (hmmalign function) and DoS are accordingly mapped.

### 2.3 Training set

Phosphorylation sites, and known kinase–substrate interactions for human, mouse, rat and yeast (respectively 9159, 1525, 763 and 3710 interactions) were downloaded from PhosphoSitePlus (Hornbeck *et al.*, 2015) and PhosphoGRID (Sadowski *et al.*, 2013). These known kinase-specific interactions were mapped on the Ensembl proteome of the selected organisms using BLAST (Camacho *et al.*, 2009). For every peptide, a window of ±7 residues surrounding the phosphorylation site was extracted. Some of the kinases involved in the known interactions had more than one kinase domain (10 in *H.sapiens*, 7 in *Mus musculus*, 3 in *Rattus norvegicus* and 1 in *Saccharomyces cerevisiae*). We therefore excluded all the interactions where the kinase had multiple kinase domains, since PhosphositePlus and PhosphoGRID do not provide information about which kinase domain is active and/or responsible for a known kinase–substrate interaction. We obtained 12 570 interactions (7809, 1306, 593 and 2862 for human, mouse, rat and yeast, respectively). The human dataset has been selected to train AKID, therefore we clustered the kinase domains in this dataset at 70% sequence identity with CD-hit (Li and Godzik, 2006). The final human training set resulted in 6654 positive interactions involving 199 kinase domains. In order to better evaluate the method performance we added an equal number of negative interactions. The dataset of negative interactions was built by associating kinases in the positive dataset with randomly-selected phosphorylated peptides from the human phosphoproteome annotated in PhosphositePlus or PhosphoGRID. A secondary dataset, of equal size, has been created with the negative interactions, involving peptides centered on S/T/Y residues, selected randomly from the proteome regardless of their phosphorylation annotation.

### 2.4 Sequence features encoding

The input of our method is composed of the kinase DoS and the residues of the target peptide converted using the orthogonal encoding. More specifically, each kinase DoS and target peptide residue is encoded into a string of 21 binary values, representing the 20 residues plus a gap position. The string position corresponding to the residues or gap is set to 1 and the other 20 characters are set to 0. Since 63 DoS and 15 peptide residues were considered, the binary input for one interaction contains 1638 values.

### 2.5 Deep neural network training

Known human KsP were used to train the deep neural network (DNN) underpinning the AKID algorithm in a 10-fold cross-validation. To find the best hyperparameters of the DNN, we performed a grid search over batch-size (values explored: 100, 200, 400, 600, 800) and number of neurons for the first (from 10 to 1010 neurons with an increase step of 200) and second hidden layer (one quarter, half and three quarters of the first layer) while maintaining fixed the default values of Maxout Function (softmax), Backpropagation Rate (0.5), Activation Function (softplus, which is a smoothed version of the rectified linear activation function), number of epochs (10) learn rate (0.5) and Backpropagation Function (resilient backpropagation). We employed an r-propagation algorithm with mean squared error function and weight normalization. At each iteration, all possible combinations of parameters were tested using receiver operating characteristic (ROC) curves and area under the curve (AUC) analysis (Supplementary Table S1A). The DNN training and test were performed using R packages *darch* (https://cran.r-project.org/web/packages/darch/index.html) and ROCR (Sing *et al.*, 2005). The hyperparameters with the best performance across our grid search are as follows: 2 hidden layers respectively of 210 and 53 neurons and a batch-size of 400.

### 2.6 Evolutionary analysis

Using our dataset of 6654 known human KsP, we inferred the orthologues of every kinase and target proteins in 45 eukaryotes. These organisms were collected from different classes, representing organisms both close and distant to *H.sapiens* (Supplementary Table S1B). We identified orthologous proteins through the OMA (Altenhoff *et al.*, 2015) database, retaining only proteins with a 1:1 orthology relationship. Matching target peptides were mapped from the global alignment [Needleman–Wunsch (Rice *et al.*, 2000)] of the human protein to its ortholog. Evolutionary distances between organisms (divergence time) were collected from TimeTree (Kumar *et al.*, 2017). Threshold scores for phosphorylations targeting serine, threonine and tyrosine (0.545, 0.517 and 0.34, respectively) were selected as those maximizing the discrimination between true KsP and false KsP recognition during the training phase (i.e. the score threshold corresponding to the intersection between the density curves of the scores of true and false KsP, Supplementary Fig. S1).

### 2.7 Prediction of KsP involved in pathogenic mutations

We collected 22 489 human genes from Ensembl (version 79). We used the R BiomaRt package (Durinck *et al.*, 2005) to retrieve the missense mutations in all the protein isoforms associated to every gene. Mutations were refined selecting only pathogenic mutations associated with a Polyphen score below 0.5 involving phosphorylatable residues. Also mutations introducing phosphorylatable residues were included in the set, which was given to AKID as input for the KsP prediction. The semantic similarity between the GO terms associated to a kinase and a target protein has been calculated through the GoSemSim package. The shortest path connecting proteins linked by predicted KsP was calculated through the physical protein–protein interaction network from STRING (Szklarczyk *et al.*, 2017): only those interactions with a score of 900 or higher in the ‘experimental’ channel were selected.

## 3 Results

We designed AKID as a method for the automatic detection of kinases and their interacting targets with no *a priori* knowledge or functional annotation. We tested the ability of the method to identify kinase domains in the proteome of *H.sapiens* and other eukaryotes. These domains were then analyzed with the Kinspect method in order to identify the residues that are responsible for the target specificity, defined as DoS. Subsequently we trained and tested a DNN by combining the DoS residues of human kinases and their known target peptides (Supplementary Fig. S2).

### 3.1 Detection of kinase and determinants of specificity

**Fig. 1.**
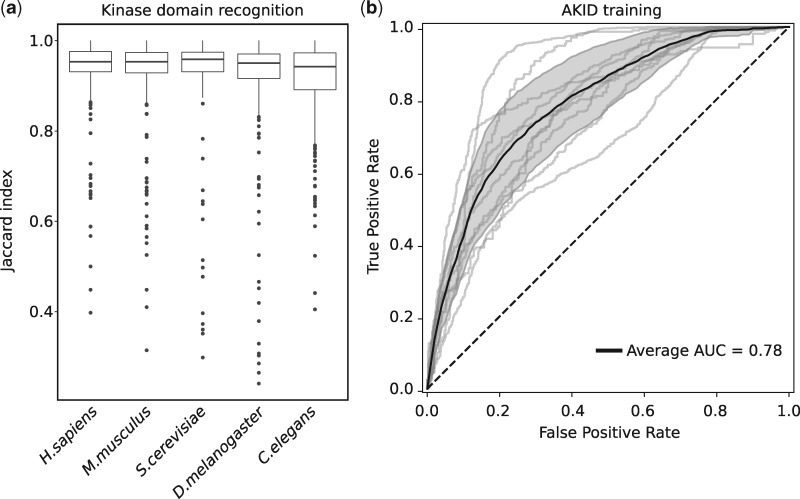
Detection of kinase domains in eukaryotes and training of AKID. **a**: Distribution of Jaccard indexes representing the overlap between the identified (via HMM) and annotated kinase domains in different eukaryotic organisms. **b**: Training of AKID through a 10-fold cross-validation. The 10 ROC curves are represented with light gray lines (summed up by a light gray area representing the 95% confidence interval), a black line represents the average performance of the method during the training. A dashed black line represents random predictions (AUC of 0.5)

We built a HMM from a manually curated multiple sequence alignment of 530 human kinase domains and used this HMM to identify 501 kinase domains in the human canonical proteome of which 496 are known and 5 have not been annotated as kinases. These kinases were identified in the human proteome (22 486 proteins, see Materials and methods) with a sensitivity of 0.94 and a specificity of 0.99 (Supplementary Table S1C). The 34 known kinases that were not identified belong to the class of the atypical kinases and, as expected, lack similarity with other kinase domains. The performance of the kinase domain detection was evaluated calculating the Jaccard index between the mapped residues of the predicted domains and the known residues belonging to the 496 kinase domains, averaging 0.93 (Fig. 1a). The majority of the kinases with a lower Jaccard index value (e.g. 15 kinases with <0.7) are again atypical kinases or kinases that do not fall in any major group and therefore can be hardly detected. The same HMM was used to detect kinases in the proteome of other organisms (*M.musculus*, *R.norvegicus*, *Drosophila melanogaster*, *Caenorhabditis elegans*, *S.cerevisiae*) reaching an average Jaccard index of 0.92 and a specificity of 0.99 on all the organisms while reaching a sensitivity of 0.92 in *M.musculus* and 0.96 in the other organisms (Fig. 1a and Supplementary Table S1D). All these results show the high reliability of AKID in the recognition of kinase domains in different eukaryotic proteomes by only using human kinase data. Finally, we map the DoS positions in all the identified kinase domains using the Kinspect algorithm. The DoS residues are then used as part of the AKID input together with the target peptide residues (see Materials and methods).

### 3.2 Training of the AKID method

The training set contains 6654 non-redundant human KsP collected from publicly available resources: 4086 phospho-serines, 1536 phospho-threonines and 1032 phospho-tyrosines, involving 199 kinases (each phosphorylating 33 sites on average). This dataset contains an equal number of negative interactions (see Materials and methods) and was used for the design and training of AKID with a DNN able to classify KsP. The 15-residues long peptide centered on the phosphosite and the 63 kinase DoS residues are then combined into an orthogonally encoded 78-residues long sequence forming the AKID input (see Materials and methods section). The DNN was trained and tested through a 10-fold cross-validation. A grid search was conducted to optimize the key parameters of the neural network (see Materials and methods section). An average AUC of 0.78 was reached during the method test (Fig. 1b and Supplementary Table S1E). Among the different kinase families tyrosine kinases performed the best with an AUC of 0.93. On the other hand the TKL family had an AUC of 0.58, reflecting the high diversity among the TKL kinases, some of which can phosphorylate both serines/threonines and tyrosines, and the fact that they are similar to tyrosine kinases but lacking their characteristic motifs (Supplementary Table S1F). Accordingly, we also observed different trends regarding the residue targeted by the phosphorylation with KsP targeting serines, threonines and tyrosines, having an AUC of respectively 0.75, 0.75 and 0.91. We also performed an additional training of the method using a secondary dataset where the negative interactions are chosen randomly from the proteome, regardless of the peptide being annotated as phosphorylated, and observed an improved performance of AKID in recognizing real KsP (AUC of 0.84). This performance highlights how the task of recognizing real KsP among phosphosites is more difficult than recognizing KsP from unphosphorylated peptides.

### 3.3 Comparison of AKID with other available methods

**Fig. 2.**
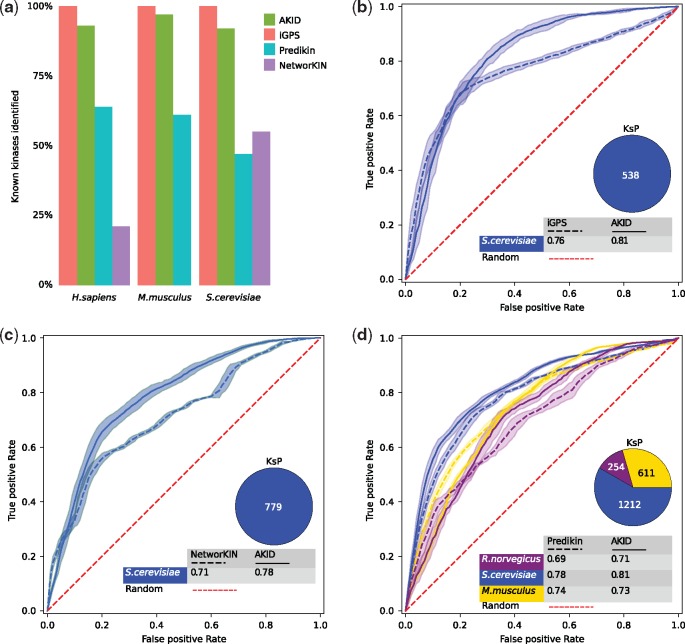
Comparison between AKID and other published methods. **a**: Coverage of known kinases (collected from KinBase) identified by different methods in three different organisms with the highest amount of known KsP. **b–d**: ROC curves, with AUC values, representing the comparison between AKID (continuous ROC curves) and the other methods (dashed ROC curves) in different organisms (ROC curves with different colors)

*H.sapiens* is the organism with the highest number of known kinase–target interactions (Hornbeck *et al.*, 2015) and it constitutes the ground of selection for the training of KsP prediction methods. Therefore, an independent validation of the method and comparison with already available methods has been run on other organisms. It has to be noted that in order to make a correct comparison between AKID and other methods a set of shared kinases, and organisms, had to be selected depending on the coverage of the other methods. We selected the iGPS (Song *et al.*, 2012), Predikin (Ellis and Kobe, 2011) and NetworKIN (Horn *et al.*, 2014) methods, as they are all similar in aims and capabilities to AKID, and built datasets with shared features for a fair comparison with AKID. We selected three different organisms, *M. musculus, R.norvegicus* and *S.cerevisiae*, to independently validate AKID and compare it to the other methods. First, we compared the kinase coverage of the different methods, testing the ability of AKID in detecting kinase domains (see Materials and methods section). iGPS covers, theoretically, all the human and yeast kinases while AKID can detect, and can predict KsP for, the vast majority of the known kinases (93, 97 and 92% of human, mouse and yeast kinases, respectively), followed by Predikin (64, 61 and 46%) and NetworKIN (21 and 55% of respectively human and yeast) (Fig. 2a). The datasets where AKID and the other methods have been compared include positive and negative KsP, selected in the same way as during the training (see Materials and methods). The interactions in the test datasets were filtered in order to avoid redundancy between test and training sets. All the KsP involving peptides with more than 50% sequence identity with any of the peptides in the human training dataset and all the kinases with more than one kinase domain were removed.

#### 3.3.1 iGPS

iGPS is a predictive algorithm that integrates information from protein–protein interaction networks to reduce the amount of false positives. The comparison between AKID and iGPS has been made using a dataset composed of 538 known KsP in *S*.*cerevisiae* involving 50 kinases shared between the two methods and for which iGPS was able to assign a score. All the tests have been repeated 10 times, each time changing the background set of negative interactions (Supplementary Table S2). AKID performed better with an average AUC of 0.81, versus the 0.76 of iGPS (Fig. 2b).

#### 3.3.2 NetworKIN

Similarly to iGPS, the NetworKIN algorithm (as part of the KinomeXplorer platform) combines a neural network to assign a phosphorylation to a specific kinase and a probabilistic protein interaction network. The shared test dataset is composed of 779 known kinase–target interactions in *S.cerevisiae* involving 39 kinases, where AKID reached an AUC of 0.78 versus 0.71 of NetworKIN (Fig. 2c and Supplementary Table S3).

#### 3.3.3 Predikin

Predikin is different from the other tested methods in that it takes into account information regarding the kinase–peptide interaction, making it similar in scope to our approach. Predikin identifies key residues of a kinase, which are those that recognize the residues around the phosphorylation sites (derived from a high-quality set of kinase-substrate complex structures); in comparison, AKID does not require spatial proximity to determine DoS residues on the kinase and thus does not require structural data. A weighted frequency matrix is then used to compare the query peptide with known target peptides of kinases with similar substrate-determining residues. We compared AKID and Predikin on three different datasets belonging to three different organisms (Fig. 2d and Supplementary Table S4): *M.musculus* (611 KsP involving 103 kinases), *R.norvegicus* (254 KsP involving 49 kinases) and *S.cerevisiae* (1212 known KsP involving 43 kinases). Apart from a comparable performance on the mouse dataset (AUC of 0.73 versus Predikin AUC of 0.74), AKID performs better than Predikin on the rat and yeast datasets (AUC of 0.81 and 0.71 versus Predikin AUC of 0.78 and 0.69, respectively).

The methods were also tested on a secondary test set, where negative interactions involving peptides centered on S/T/Y residues were randomly selected form the proteome regardless of their phosphosylation annotation. We observed an improvement in the performance of every method, with AKID outperforming the other methods, revealing how every method was able to better identify real KsP among unmodified peptides instead of phosphorylated peptides (Supplementary Table S5A). Although AKID and the compared methods have been trained on human KsP and tested on different organisms we compiled a human dataset composed of KsP, 374 real KsP and an equal number of negative interactions (Supplementary Table S5B), that have been curated with the same criteria as the other datasets (see Materials and methods), recently collected in PhosphoSite Plus in the latest release (May 2018). We measured an AUC of 0.8 against the 0.78 of Networkin and an AUC of 0.76 against the 0.72 of iGPS (the differences in the performance of AKID are due to the slightly different versions of the test sets, which are dependent on the kinase coverage of each method). Considering these results we can conclude that the integration of the information of DoS residues on the kinase domain and the target peptide leads to an improved encoding of the kinase specificity rules and to an improved identification of KsP in different organisms.

### 3.4 Evolutionary conservation of known human interactions in orthologous proteins

In order to investigate and explore the different AKID performances among the different kinase groups we analyzed the conservation of 6654 known human KsP across 45 eukaryotes. This allowed us to analyze the evolution of the KsP in different organisms. We searched for the ortholog of each human KsP in the other organisms, requiring that both the kinase and the kinase-target orthologs could be identified with a 1:1 orthology relationship in order to reduce complexity (see Materials and methods). The amount of human KsP identified orthologs ranges from the 60% among mammalian organisms to the nearly 50% in reptiles, amphibians and lobe-finned fishes, to the 0.1 and 0.3% in *C*.*elegans* and *S*.*cerevisiae*, respectively (Supplementary Fig. S3A). The different percentages of mapped KsP are not only due to actual evolutionary gain or loss of the KsP but also to incomplete annotation or complex orthology relationships (as can be seen especially in yeast). As expected, the fraction of conserved orthologous KsP decreases with the increasing evolutionary distances from *H*.*sapiens* (Supplementary Fig. S3B, red line). We divided the human KsP into kinase groups and measured the conservation of the phosphorylations of each group during evolution. Growth, stress-response kinases and cell cycle-related kinases of the CMGC group and kinases of the AGC group (cytoplasmic kinases regulated by second messengers) are similarly conserved, as the average of the kinases (Supplementary Fig. S3B, blue and gold line, respectively). Phosphorylations related to the STE group, responsible for the activation of MAPK family, are poorly conserved (50–60% from mammalian through ray-finned fishes), suggesting that the activation of the MAPK family is less constrained by evolution with class-specific regulation mechanisms (Supplementary Fig. S3B, green line). On the other hand, we observed a high degree of conservation (>90%), from *H.sapiens* to Insecta, of KsP involving tyrosine kinases (TK family, Supplementary Fig. S3B, purple line). It has been observed that this group of kinases evolved recently (Miller, 2012) and this may be one of the possible explanations for the high conservation of KsP from *H.sapiens* to Insecta, also explaining the high performance of AKID in recognizing KsP targeting tyrosine residues. This analysis gives an evolutionary perspective on the reasons why AKID shows a variable ability in capturing the target specificity rules that characterize different groups of kinases, as we have observed during the training of the method.

### 3.5 Large-scale prediction of pathogenic alteration of phosphorylation sites

The method proposed in this work can be applied to large datasets with the aim of discovering novel KsP in particular cellular conditions and providing biological insights of clinical relevance. We collected 22 057 missense pathogenic mutations from Ensembl and selected 5714 pathogenic missense mutations changing serines, threonines or tyrosines into non-phosphorylatable residues or changing other residues into phosphorylatable ones. We did not consider mutations affecting the residues surrounding the phosphorylation sites, as estimating the change of affinity of a kinase for a mutated peptide is beyond the scope of this work. This kind of mutations can have a range of different effects on the protein structure and function. Since we aim at selecting only those mutations disrupting the phosphorylation-related events, with no harmful consequences on the protein structure, we discarded mutations for which Polyphen (Adzhubei *et al.*, 2013) predicted a strong impact on the protein structure (Polyphen score higher than 0.5). The peptides hosting the remaining 1398 missense mutations were analyzed with AKID against the human kinases, resulting in 1589 unique novel predicted KsP with high score (3787 when accounting for identical phosphopeptides in different protein isoforms), targeted by pathogenic missense mutations that are remodeling phosphorylation-related signaling pathways (Supplementary Table S1G).

In order to provide an external validation of these predictions we used GOSemSim (Yu *et al.*, 2010), a method able to assess the semantic similarity of GO terms between two proteins. In fact, we observed that a predicted interaction involved a kinase and a target protein that are more likely to have similar biological functions, and to share same or similar cellular localizations, more than a pair of random proteins (Mann–Whitney test *P *=* *3.4×10^−4^ for biological function and *P *=* *3.2 ×10^−6^ for cellular component). We calculated the shorted path connecting the kinase and their predicted targets in the human interactome (Szklarczyk *et al.*, 2017); again we observed the kinase and their predicted targets to be significantly closer (Mann–Whitney test *P* < 0.05) than random pairs of proteins. We think that this list contains novel kinase–target interactions of high clinical relevance. It represents a valuable resource for follow-up studies on how disease states are able to rewire the phosphorylation network between kinases and their targets.

### 3.6 AKID web server

We have developed a web server that allows an easy access to the AKID method. The server accepts kinases and target proteins as user input and scores the target residues most likely to be phosphorylated by the input kinases. Kinases and targets can be provided by the user in FASTA format or as UniProt ids, either in the form text area or by uploading a text file. The server performs the additional step of checking if each of the entered sequences is recognized as a kinase by the algorithm and, if so, identifies the DoS in the kinase sequence. If more than one kinase domain is found in a protein, the server will treat the domains individually and identify their respective DoS.

As an alternative, entire kinomes from 55 organisms can be selected; in this case kinase DoS are pre-calculated and stored. The server can process up to 500 kinase-target couples, or up to 10 targets for a selected kinome in a single job. Once a job is started, a score for each DoS-target residue couple is computed. A visual output is provided together with a tab-delimited csv file that associates each couple with a probability score. If the input data comprises 1 kinome and 1 target, or up to 50 kinase-target couples, the server provides access to more tools to visualize, browse and sort the output data. The residue and kinase data views allow to easily find the kinase(s) most likely to phosphorylate a specific residue, or the residue(s) most likely to be the target of a specific kinase, respectively. Details on the available views can be found in the ‘about’ page on the web server. For large-scale predictions in which the amount of data to be analyzed exceeds the acceptable load for the web server, or a more custom analysis pipeline is desired, we provide a standalone software that allows the implementation of the AKID method on a Linux machine that can be found in the AKID web server at the following address: http://akid.bio.uniroma2.it.

## 4 Discussion

Despite the thousands of phosphorylation sites discovered in large-scale experiments every year, the information about the responsible kinases is missing for the vast majority of them. A number of methods for the prediction of KsP have been developed to fill this gap. The novelty of AKID stands in the integration of information characterizing both the target peptide and the residues that are determining the specificity of a kinase, without requiring structural information or *a priori* annotation of kinases in the proteome to be analyzed. AKID has been trained on a curated set of 6654 known human KsP events, reaching an average AUC of 0.78 in a 10-fold cross-validation test. We compared AKID with other methods with similar aims, capabilities and approaches on independent test sets in different organisms. In terms of kinase coverage AKID is able to detect the vast majority (>90%) of the kinome in different organisms while others reached lower coverage (e.g. Predikin has its highest coverage in *H.sapiens* with 64% of the kinases) or were not able to identify known KsP for many kinases. AKID outperformed the other methods, even those that were including protein–protein interaction data into their predictive models. In terms of kinase coverage we observed that atypical kinases, nucleoside diphosphate kinases (NDK group) and other unconventional kinases could not be recognized through the curated HMM we used, therefore not allowing the prediction of their determinant residues used by AKID for the prediction of KsP. Although they represent a small fraction of the whole kinome not all of the members of these groups have shown kinase activity. We noticed a differential performance of the method depending on the kinase group considered. AKID could not successfully identify KsP associated to the TKL group (AUC of 0.58); this kinase group is very diverse and although its kinases are all similar to tyrosine kinases they lack the typical functional motifs (Manning *et al.*, 2002b). Moreover some of its members are dual-specificity kinases and can phosphorylate tyrosines, serines and threonines. This suggests that the specificity rules of this class cannot be easily represented and identified through the AKID neural network. On the other hand we noticed a very high performance of AKID in correctly recognizing human KsP involving tyrosine kinases (AUC of 0.93). Intrigued by the different scoring of phospho-tyrosines when compared to KsP involving phospho-serines and phospho-threonines (both with an AUC of 0.75), we analyzed the evolutionary component of the specificity of these phosphorylations through the conservation of known human kinases-specific phosphorylation and their orthologs in 45 eukaryotes. Again we found a peculiar high conservation of tyrosine phosphorylation from *H.sapiens* to Insecta. This might be explained by the observation that the TK group evolved later than other kinases (Miller, 2012) and has very clear and conserved specificity determinant in both the kinase and target peptide sides. Finally, we demonstrated the usefulness of the method by scanning a whole set of phosphorylatable residues hit by pathogenic mutations in the human proteome and deriving a list of predicted KsP with high score. For this reason, we believe that AKID will be of great value for the prioritization of KsP with a potentially high clinical relevance.

## Supplementary Material

Supplementary Figure S1

Supplementary Figure S2

Supplementary Figure S3

Supplementary Materials

Supplementary Table S1

Supplementary Table S2

Supplementary Table S3

Supplementary Table S4

Supplementary Table S5
