# Supplementary figures and images for: Kinome-wide identification of phosphorylation networks in eukaryotic proteomes
Source: Bioinformatics. 2018 Jul 17;35(3):372–9. doi: 10.1093/bioinformatics/bty545 (PMC6361239; doi:10.1093/bioinformatics/bty545)

Phospho-Serines

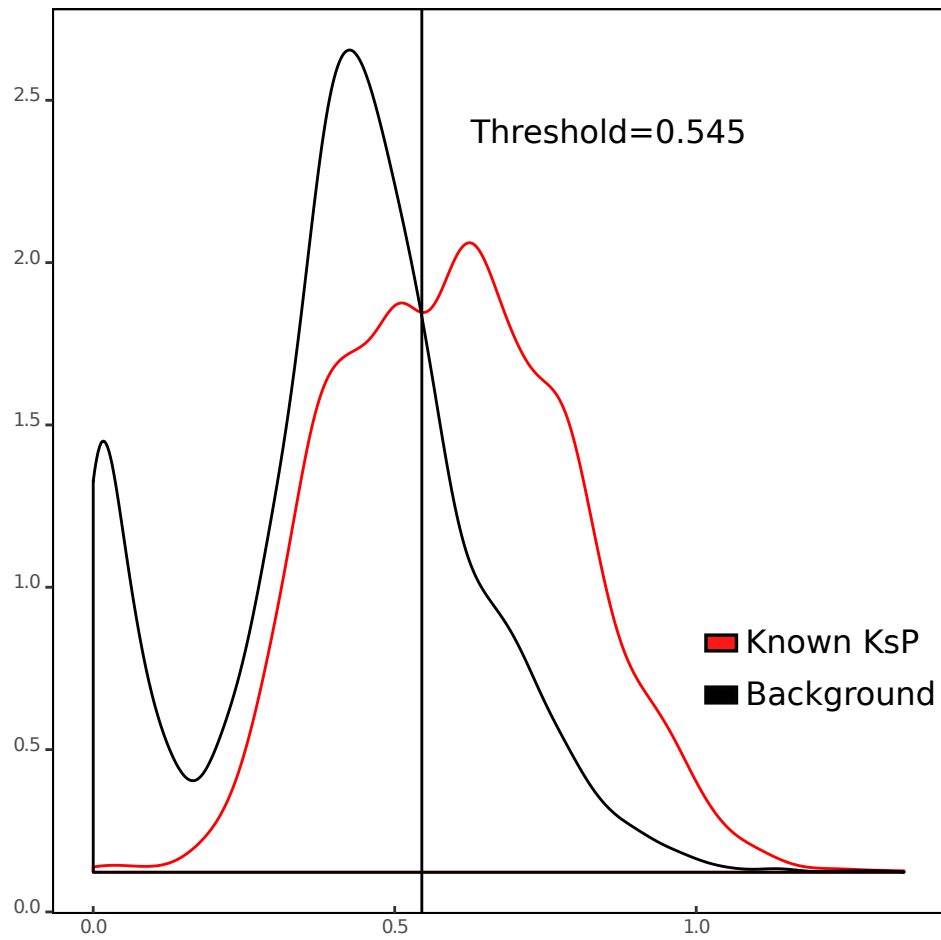

Phospho-Threonines

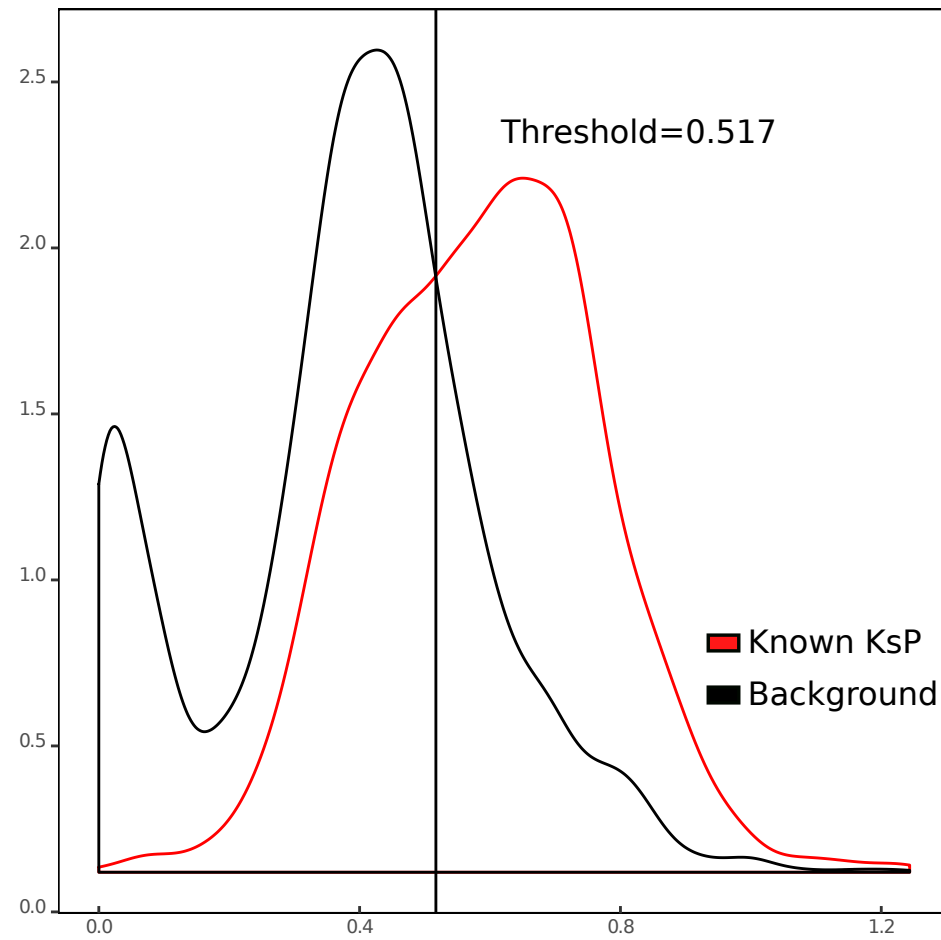

Phospho-Tyrosines

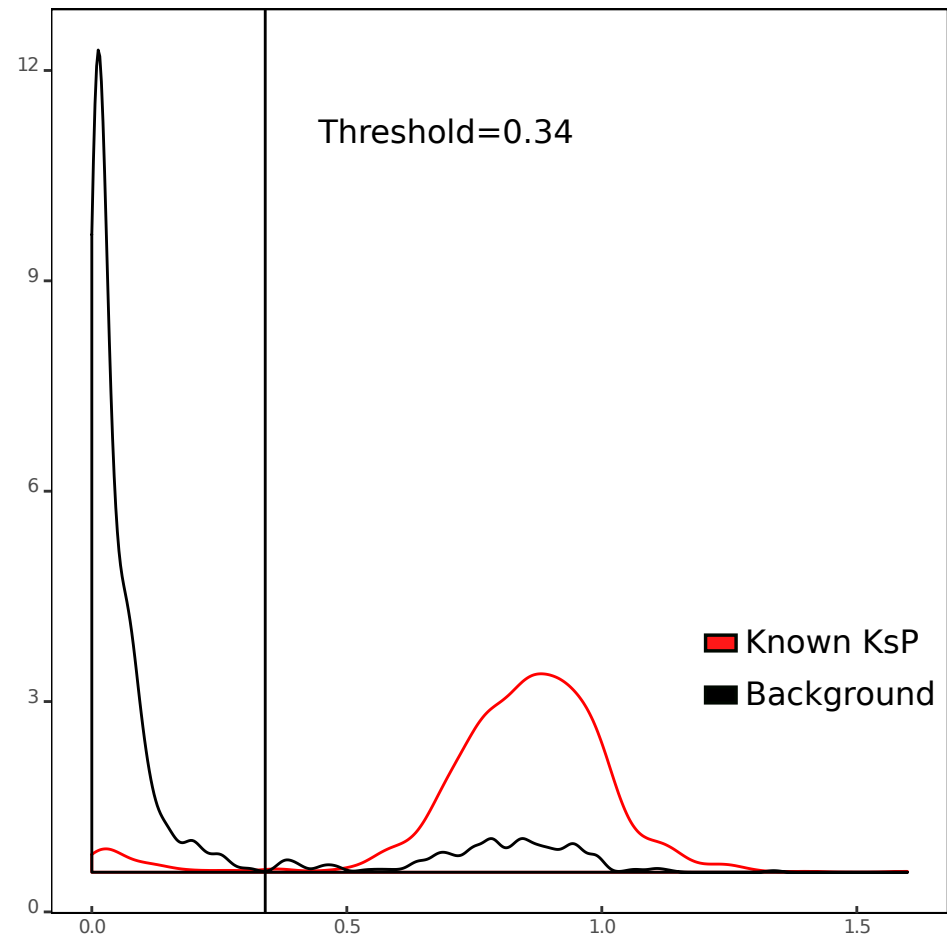

Supplement: Supplementary Figure S1 [file bty545_supplementary_figure_s1.pdf]

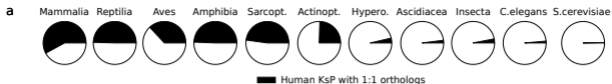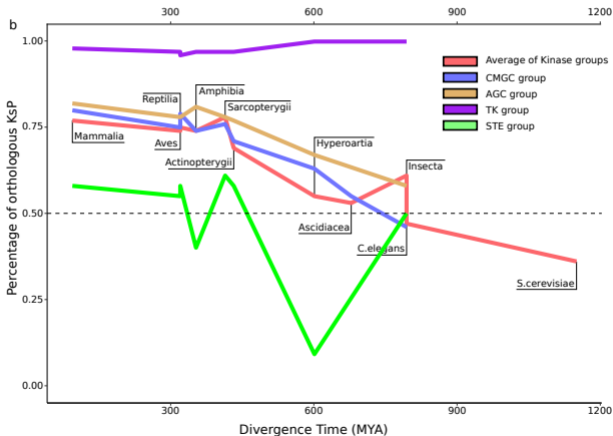

Supplement: Supplementary Figure S3 [file bty545_supplementary_figure_s3.pdf]
