# Supplementary material for: Kinome-wide identification of phosphorylation networks in eukaryotic proteomes
Source: Bioinformatics. 2018 Jul 17;35(3):372–9. doi: 10.1093/bioinformatics/bty545 (PMC6361239; doi:10.1093/bioinformatics/bty545)
Supplement: Supplementary Figure S2 [file bty545_supplementary_figure_s2.pdf]

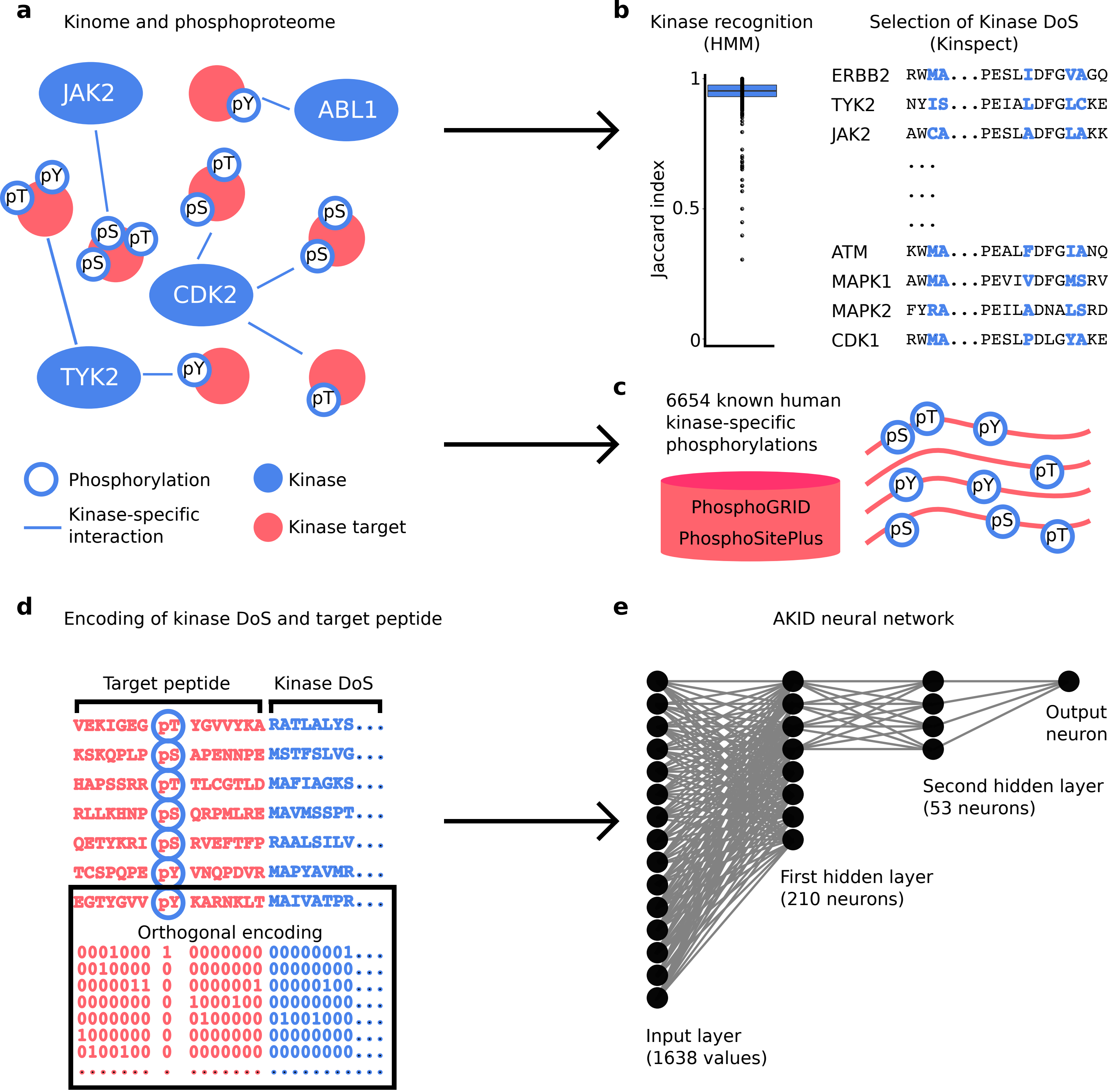
a
Kinome and phosphoproteome
JAK2
ABL1
CDK2
TYK2
pY
pT
pS
Phosphorylation
Kinase
Kinase-specific interaction
Kinase target
b
Kinase recognition (HMM)
Jaccard index
1
0.5
0
Selection of Kinase DoS (Kinspect)
ERBB2 RWMA...PESLIDFGVAGQ
TYK2 NYIS...PEIALDFGLCKE
JAK2 AWCA...PESLADFGLAKK
...
ATM KWMA...PEALFDFGIANQ
MAPK1 AWMA...PEVIVDFGMSRV
MAPK2 FYRA...PEILADNALSRD
CDK1 RWMA...PESLPDLGYAKE
c
6654 known human kinase-specific phosphorylations
PhosphoGRID
PhosphoSitePlus
d
Encoding of kinase DoS and target peptide
Target peptide
Kinase DoS
VEKIGEG pT YGVVYKA RATLALYS...
KSKQPLP pS APENNPE MSTFSLVG...
HAPSSRR pT TLCGTLD MAFIAGKS...
RLLKHNP pS QRPMLRE MAVMSSPT...
QETYKRI pS RVEFTFP RAALSILV...
TCSPQPE pY VNQPDVR MAPYAVMR...
EGTYGVV pY KARNKLT MAIVATPR...
Orthogonal encoding
0001000 1 0000000 00000001...
0010000 0 0000000 00000000...
0000011 0 0000001 00000100...
0000000 0 1000100 00000000...
0000000 0 0100000 01001000...
1000000 0 0000000 00000000...
0100100 0 0000000 00000000...
e
AKID neural network
Input layer (1638 values)
First hidden layer (210 neurons)
Second hidden layer (53 neurons)
Output neuron
