# Supplementary material for: Kinome-wide identification of phosphorylation networks in eukaryotic proteomes
Source: Bioinformatics. 2018 Jul 17;35(3):372–9. doi: 10.1093/bioinformatics/bty545 (PMC6361239; doi:10.1093/bioinformatics/bty545)
Supplement: Supplementary Materials [file bty545_supplementary_materials.docx]

**Supplementary Materials**

**Supplementary Figure S1:** Treshold used in the evolutionary analysis to label predicted orthologous KsP for each phosphorylatable residue. The distribution of the scores for known KsP is colored in red, while the score distribution of the background, negative, KsP is colored in black. Threshold score is determined at the intersection of the distribution curves.

**Supplementary Figure S2:** Illustration of the methodology underpinning AKID. **A**: Schematic view of a portion of the network connecting the nearly 500 human kinases (blue) and their target (red) in the proteome of a cell. **B**: Detection of the kinase domains among the proteins of the proteome through a curated Hidden Markov Model generated from nearly 500 human kinase domains. Kinase domains can be detected with high sensitivity (higher than 0.92) and specificity (0.99) in different organisms, with a very high overlap between the real kinase domains and the one identified through the HMM (high Jaccard index of the overlapping residues). The identified domains are then scanned with the Kinspect methodology (Creixell, Palmeri, *et al.*, Cell 2015) in order to find the 63 residues that are important for the target specificity (colored in blue), termed Determinants of Specificity (DoS). **C**: 6654 known human Kinase-specific Phosphorylations (KsP) are collected from PhosphoSitePlus and PhosphoGRID . **D**:. The DoS residues of the kinase and the 15-residue long peptide centered on the KsP are then combined into a 78-residue long sequence which is orthogonally encoded, as described in the Methods, into the input of the neural network **E**: Structure of the deep neural network used by AKID. The first input layer of the network is constituted by the 1638 values corresponding to the encoded input sequence. This is followed by two hidden layers (210 and 53 neurons each) and the output layer which is represented by a unique neurons. The output is a continuous value which can be used to discriminate between true and false, or background, KsP (which is the value used to plot the ROC curves and to calculate the AUC of the various performances).

**Supplementary Figure S3:** Mapping and conservation of orthologous KsP**.** **A**: 6654 human KsP were mapped, where possibile, through a 1:1 orthology relationship in 45 Eukaryotes divided into 11 Classes. The percentage of mapped KsP in each Class is represented by pie charts. **B**: Analysis of the conservation of the mapped orthologous KsP in 45 Eukaryotes. The X axis represents the divergence time between a Class and *H.sapiens* (in million years, X axis) while the Y axis represents the average percentage of orthologous KsP conserved in each Class of organisms (red line). A breakdown of this percentage by kinase groups is represented by the blue (CMGC kinase group), orange (AGC kinase group), purple (TK group) and green (STE kinase group) lines.

**Supplementary Table S1:** Table **S1A** contains the results of the grid search of the deep neural network, explored values for the size of the first, second layer and batch size are reported together with the AUC. Table **S1B** contains the list of the 45 Eukaryotes analyzed in the evolutionary analysis, annotated with species, class, divergence from *H.sapiens* (in million years) and number of human KsP mapped through orthology. Table **S1C** contains the list of detected (with calculated Jaccard index showing the overlap between mapped and predicted kinase domain) and undetected kinases using the HMM of human kinases. Table **S1D** contains the performance of the kinase domain detection using the HMM in different proteoms, reporting Sensitivity and Specificity with the number of True Positives, False Positives, True Negatives, False Negatives in each organism. Table **S1E** shows the performance of the training phase of the method with the scores associated to each real or background KsP (kinase, target protein and phosphorylated site). Table **S1F** contains the breakdown, by kinase group and residue type, of the performance (AUC) of the method during the training phase. Table **S1G** contains the prediction of novel KsP in the human proteome in sites hit by pathogenic mutations (both in the case of removal or creation of phosphorylatable sites).

**Supplementary Table S2:** Performance of AKID and iGPS on the same dataset of known KsP in yeast. 10 different prediction runs have been made changing the background KsP.

**Supplementary Table S3:** Performance of AKID and Networkin on the same dataset of known KsP in yeast. 10 different prediction runs have been made changing the background KsP.

**Supplementary Table S4:** Performance of AKID and Predikin on the same dataset of known KsP in yeast, rat and mouse. 10 different prediction runs have been made changing the background KsP in each organism.

**Supplementary Table S5:** Table **S5A** contains the performance (expressed as AUC) of AKID and the compared methods using alternative datasets where the selected negative interactions (KsP) target random peptides, in the proteome of the organism, centered on S/T/Y residues regardless of their phosphorylation annotation. Table **S5B** contains the dataset of 748 human interactions used to compare AKID with the other methods (the dataset is repeated 10 times, with the negative interactions changing each time).
